# Rapid cancer diagnosis using deep learning–powered label-free subcellular-resolution photoacoustic histology

**DOI:** 10.1126/sciadv.adz1820

**Published:** 2025-11-21

**Authors:** Byullee Park, Rui Cao, Yilin Luo, Cindy Liu, Yushun Zeng, Yide Zhang, Qifa Zhou, Samuel Davis, Massimo D’Apuzzo, Lihong V. Wang

**Affiliations:** ^1^ Caltech Optical Imaging Laboratory, Andrew and Peggy Cherng Department of Medical Engineering, California Institute of Technology, Pasadena, CA, USA.; ^2^ Alfred E. Mann Department of Biomedical Engineering and Ophthalmology, Viterbi School of Engineering, University of Southern California, Los Angeles, CA, USA.; ^3^ Department of Pathology, City of Hope Beckman Research Institute and Medical Center, Duarte, CA, USA.

## Abstract

Traditional hematoxylin and eosin staining in formalin-fixed paraffin-embedded sections, while essential for diagnostic pathology, is time-consuming, labor intensive, and prone to artifacts that can obscure critical histological details. Label-free ultraviolet photoacoustic microscopy (UV-PAM) has emerged as a promising alternative, offering fast histology-like images without the need for traditional staining and excessive tissue preparation. However, current UV-PAM systems face challenges in achieving the high spatial resolution required for detailed histological analysis and diagnosis. To address this, we developed a subcellular-resolution UV-PAM (SRUV-PAM) system with a 240-nanometer resolution, enabled by the integration of a high numerical aperture (NA) objective lens (NA = 0.64) and the precise piezo actuators for fine scanning control. This configuration allows visualization of detailed nuclear structures. In addition, we demonstrated virtual staining of SRUV-PAM images via cycle-consistent generative adversarial networks and diagnosis of malignant and benign tumors in liver tissues via densely connected convolutional networks DenseNet-121, achieving an area under the receiver operating characteristic curve of 0.902.

## Teaser

Deep learning and label-free photoacoustics combine to enable rapid, subcellular-resolution cancer diagnosis without delay.

## INTRODUCTION

Histopathological examination through hematoxylin and eosin (H&E) staining has been the cornerstone of diagnostic pathology for decades (*1*, *2*). This method provides crucial insights into the phenotype and density of cells and the overall tissue structure, allowing pathologists to distinguish between healthy and neoplastic tissues. However, despite its widespread use and reliability, traditional H&E staining presents substantial tissue preparation time and manual effort, which can hinder rapid diagnostic workflows. In addition, the interpretation of stained tissues depends heavily on the expertise of the pathologist, making the process subjective in some cases and challenging for standardization (*3*–*5*).

Various label-free optical imaging modalities have been developed to support histological diagnosis without conventional staining, each offering unique advantages and trade-offs. Stimulated Raman scattering microscopy enables chemical contrast based on vibrational signatures and offers rapid imaging over large areas but typically provides limited spatial resolution and slow imaging speed and often requires complex laser systems (*6*). Optical coherence tomography and optical coherence microscopy provide fast, depth-resolved cross-sectional imaging using scattering contrast, but they generally lack the molecular specificity and lateral resolution necessary for identifying subcellular nuclear features (*7*). Microscopy with ultraviolet (UV) surface excitation enables rapid surface imaging of fresh tissues but suffers from shallow imaging depth and requires labeling for visualization (*8*). Light-sheet microscopy achieves high-resolution volumetric imaging with minimal photodamage but often involves sample clearing or fluorescent labeling steps that complicate clinical translation (*9*). Despite their respective strengths, most of these technologies fall short in providing subcellular-resolution, label-free nuclear contrast that is directly comparable to conventional H&E histology.

Recently, UV photoacoustic microscopy (UV-PAM) has garnered attention as a tool capable of overcoming the limitations of traditional H&E staining and providing histology-like or virtual histology images without staining (*10*–*15*). PAM is based on the photoacoustic (PA) effect, where pulsed laser light is absorbed by tissues, causing rapid thermoelastic expansion that generates ultrasonic (US) waves. These US waves are then detected and used to construct subcellular-resolution images of the optical absorption distribution in the tissue, providing valuable functional and molecular information (*16*–*22*). Notably, the high optical absorption of nucleic acids in cell nuclei in the UV spectrum allows UV-PAM to image them in biological tissues (*23*–*25*). This unique capability enables the detailed visualization of cellular structures for histopathology without the need for fixation, processing, embedding, sectioning, and staining, preserving the natural state of the tissues and preventing tissue loss and potential procedure artifacts associated with the standard histology. Recent advancements of UV-PAM–based histopathology have shown its great potential in clinical settings. In 2017, Wong *et al.* (*10*) successfully acquired histology-like PA images from human breast tissues and identified diagnostic features for breast cancer. In 2020, Haven *et al.* (*11*) introduced UV PA remote sensing (UV-PARS)–based histopathology . This noncontact approach suggests the potential for better preservation of samples or simplification of sample preparation. In 2021, Baik *et al.* (*12*) developed a high-speed reflection-mode UV-PAM using a one-axis micro-electro-mechanical systems scanner. This technique allowed for the PA acquisition of cancer tissue, followed by pseudocoloring to create H&E-like images. In 2022, Cao *et al.* (*13*) successfully applied deep learning–based virtual staining, demonstrating clinician-readable PA histological imaging. In addition, they implemented three-dimensional (3D) contour scanning to achieve high-resolution imaging of thick, unprocessed samples with uneven surfaces. Subsequently, Martell *et al.* (*14*) integrated deep learning–based virtual staining into UV-PARS and conducted a blinded pathologist reader study, confirming its diagnostic potential. In 2024, Kim *et al.* (*15*) developed a transparent ultrasound-based UV-PAM, enhancing resolution and validating virtual histopathology in thick, unprocessed samples. In addition, studies using conventional H&E images have shown that deep learning models can achieve strong diagnostic performance even with limited training data, especially when leveraging transfer learning (*26*–*29*).

Despite its potential, current UV-PAM systems are limited in spatial resolution due to challenges in designing high numerical aperture (NA) optics for UV wavelength and combining it with the ultrasound transducer (UT) in subcellular-resolution PAM configuration. In the meantime, comprehensive histopathological analysis often requires an imaging system with a resolution in the range of 200 to 300 nm to see cell nuclei details (*30*–*32*). Recent advances in pathology also highlight a growing demand for higher-resolution imaging to capture subtle subcellular features, reinforcing the importance of continued development toward systems capable of bridging conventional histology and emerging high-resolution modalities (*33*, *34*). Thus, existing UV-PAM systems with insufficient resolution limit their effectiveness for detailed histopathological analysis and comprehensive diagnosis. To address this challenge, we developed a piezo actuator–based subcellular-resolution UV-PAM (SRUV-PAM) with deep learning–powered virtual histology and tumor diagnosis. We demonstrated histology-like images of human kidney and liver tissues via SRUV-PAM with a 240-nm resolution, which is comparable to the resolution of conventional histopathological imaging. After obtaining these images, we applied deep learning–based virtual staining using cycle-consistent generative adversarial networks (CycleGAN) to enhance interpretability, which allows pathologists to understand and interpret the virtually stained PA images without additional training. Furthermore, for diagnostic evaluation, we used a modified DenseNet-121 model to differentiate malignant and benign liver tissues, confirming the diagnostic value of our system. Our SRUV-PAM system overcomes the limitations of previous UV-PAM technologies, providing superior imaging quality and enabling more detailed and accurate histopathological analysis. This advancement not only enhances the capabilities of UV-PAM but also broadens its potential applications in biomedical research and clinical diagnostics.

## RESULTS

### Deep learning–powered SRUV-PAM for histopathology and diagnosis

**Fig. 1.**
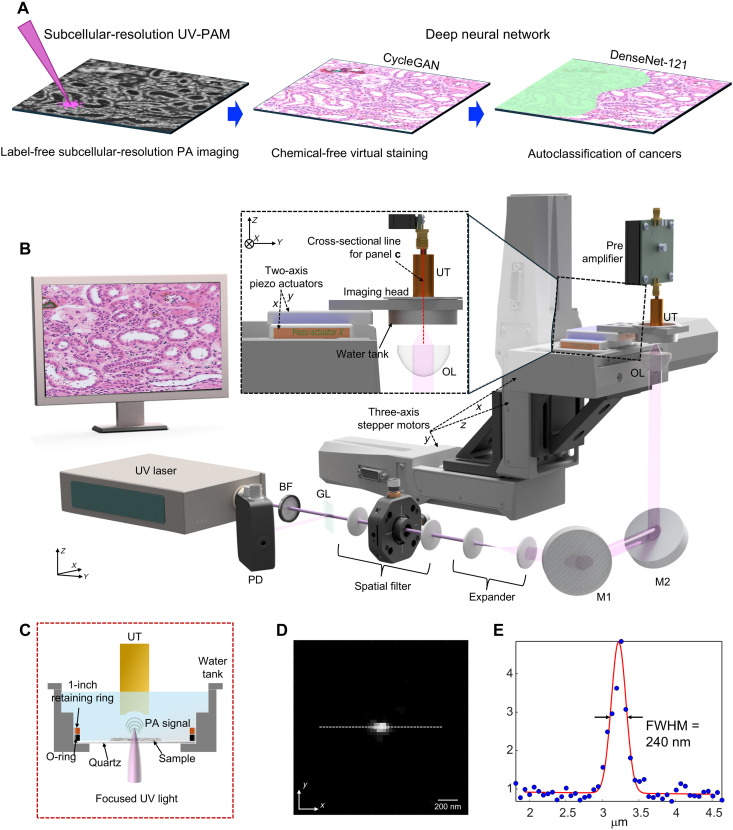
Strategy and system characteristics of deep learning–powered label-free SRUV-PAM for histopathology and cancer diagnosis. (**A**) A three-step strategy includes the following: (i) Label-free subcellular PA imaging using SRUV-PAM to acquire PA images of tissue slices, (ii) chemical-free virtual staining using CycleGAN to generate virtually stained histology images, and (iii) automated cancer classification leveraging the DenseNet-121 deep neural network to differentiate malignant and benign regions. (**B**) Detailed system schematic of SRUV-PAM. The system consists of a UV laser source, beam expansion, and filtering components, and an imaging stage with three-axis stepper motors. The inset illustrates the connection between the two-axis piezo actuators and the imaging head. OL, objective lens; GL, glass; M, mirror; BF, optical beam filter. (**C**) Cross-sectional view of the imaging head, acquired along the red dashed line in (B). (**D**) PA image of a 100-nm gold nanoparticle. (**E**) Line profile obtained from the dashed line indicated in (D). The lateral resolution of the system is measured to be ~240 nm based on the full width at half maximum (FWHM) of this line profile.

First, SRUV-PAM used a 266-nm wavelength nanosecond pulsed laser for optical absorption contrast of DNA/RNA (Fig. 1, A and B) (*35*). The cross section of the imaging head, including a water tank and a tissue slice sample fixed on a 200-μm-thick quartz slide, was irradiated with a tightly focused laser using a high NA (0.64) lens, which is the highest used to date for UV-PAM. An O-ring was used to prevent water leakage. A 2.54 cm retaining ring was used to securely fix the O-ring and quartz. At the opposite position of the optical focus, a UT with a center frequency of 40 MHz collected the PA signal, and the collected signal was digitized at a sampling rate of 500 MHz and stored on a host computer. A 3D stepper motor scanning stage system (in *XYZ* planes) and a 2D piezo actuator scanning stage (in *XY* planes) are combined to simultaneously satisfy the requirements of a large field of view and subcellular-resolution raster scanning. Because both modules are integrated within a single platform, the system inherently enables both low-resolution survey and high-resolution imaging without the need for hardware switching or objective exchange. A sample holder containing the tissue slice sample was connected to the piezo actuators, which imaged a 30 μm–by–30 μm area at a time with 1000 by 1000 pixels. The system was further optimized through hardware-software codesign. A high-precision piezo-driven stage was used for *x*-*y* scanning at nanometer-level precision (30 nm per pixel). To correct for nonlinear motion distortions arising during high-speed scanning—particularly along the fast axis—we implemented a software-based compensation algorithm, which substantially improved the geometric accuracy of reconstructed images. The corrected carbon fiber PA image exhibits uniform signal representation across the entire scanning region (fig. S1). This correction was essential for preserving spatial fidelity in subcellular-resolution imaging. The lateral resolution of SRUV-PAM was measured to be 240 nm by imaging a 100-nm-diameter gold nanoparticle (Fig. 1, C and D). After accounting for the sample size effect, the intrinsic system resolution was estimated to be 218 nm, which is close to the theoretical diffraction-limited resolution of 208 nm (calculated using the 0.5λ/NA criterion at λ = 266 nm, NA = 0.64). The motor scanners connected to the sample holder and piezo actuators covered an area up to 25 mm^2^ with a step size of 156 nm. The *z*-axis stage of the motorized stage allowed precise positioning of the sample in optical focus. When observing the detailed structure of single cells, we used piezo actuator–based imaging to achieve the best resolution. For deep learning–based PA virtual staining images, we used stepper motor scanners to achieve the large field of view, respectively.

**Fig. 2.**
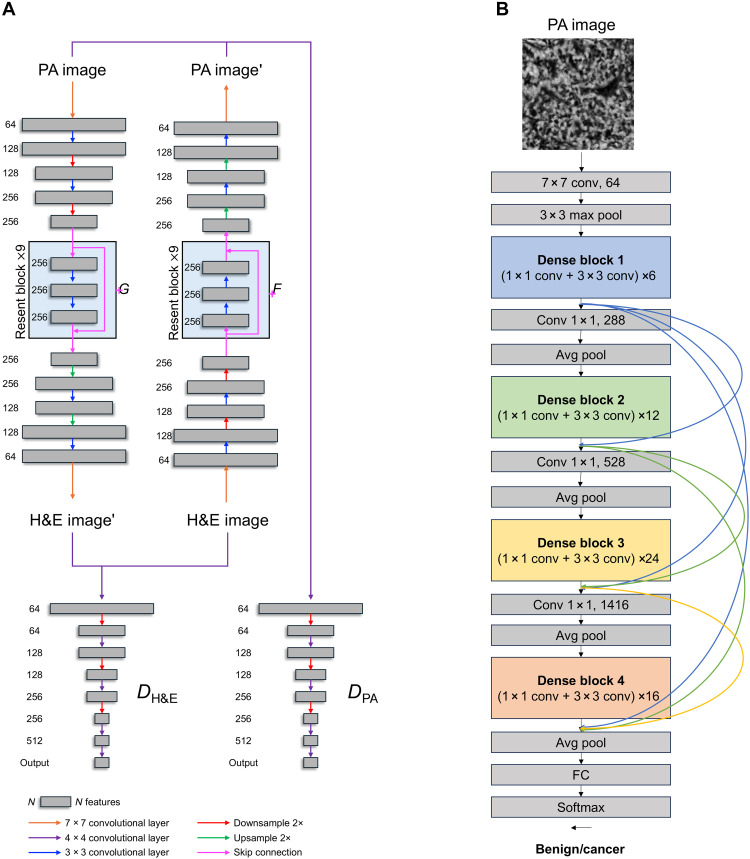
Deep learning architectures for virtual histology and cancer classification. (**A**) CycleGAN-based virtual staining for histopathology and (**B**) DenseNet-121–based deep learning model for automated cancer classification. Avg, average; FC, fully connected layer; Conv, convolutional layer.

Second, we used CycleGAN architecture for virtual H&E staining, as depicted in Fig. 2A, to facilitate the unsupervised image mapping from the UV-PAM domain (PA) to the H&E domain (HE). Two generators were trained to perform transformations *G*: PA → HE and *F*: HE → PA, while two discriminators, *D*_PA_ and *D*_HE_, evaluate the authenticity of images generated by *G* and *F*, respectively. The adversarial loss for discriminators drives them to optimize their ability to differentiate between real and generated images

$$l^{D_{\text{HE}}}=D_{\text{HE}}{[G\left(\text{PA})\right]}^{2}+{\left[1–D_{\text{HE}}\left(\text{HE}\right)\right]}^{2}$$ (1)

$$l^{D_{\text{PA}}}=D_{\text{PA}}{\left[G\left(\text{HE}\right)\right]}^{2}+{\left[1–D_{\text{PA}}\left(\text{PA}\right)\right]}^{2}$$ (2)

The cycle consistency loss ensures that an image translated from one domain should closely resemble the original image

$$\begin{array}{c} l^{G}={\left\{1-\left[G\left(\text{PA}\right)\right]\right\}}^{2}+\{{1-D_{\text{HE}}\left[F\left(\text{HE}\right)\right]\}}^{2}+\\ \lambda |F\left[G\left(PA\right)\right]-\mathrm{PA}|+\lambda |G\left[F\left(\text{HE}\right)\right]-\text{HE}| \end{array}$$ (3)

Details of the network architecture can be found in Materials and Methods. Our training dataset included SRUV-PAM images and H&E images of kidney and liver specimens. The kidney dataset consists of 13 and 45 images with 2000 by 2000 pixels for UV-PAM and H&E histology, respectively. The liver dataset has 46 and 20 images with 4000 by 4000 pixels for UV-PAM and H&E histology, respectively. These images were randomly cropped into smaller patches of 512 by 512 pixels before data augmentation. The training used the Adam solver with a batch size of 4 and an initial learning rate of 0.0002, decaying to zero over 100 epochs.

Third, the ability of SRUV-PAM to aid clinical diagnosis was demonstrated using a deep learning classification model. Grayscale UV-PAM images were used to train a supervised deep learning model for automated classification of benign versus cancer sections. The diagnosis model consists of a modified dense convolutional network (DenseNet-121; Fig. 2B), which takes in 300 μm–by–300 μm sections of UV-PAM images as input. DenseNet-121 uses dense blocks for feature extraction, with each block containing multiple convolutional layers and channel-wise concatenation of feature maps between blocks. This densely connected feed-forward structure requires fewer model parameters while improving the flow of information within the network, allowing DenseNets to learn efficiently even with limited training data. More details of the classification model architecture can be found in Materials and Methods. The final output of the classifier is a binary prediction of benign or cancer.

### Label-free SRUV-PAM with virtual staining

**Fig. 3.**
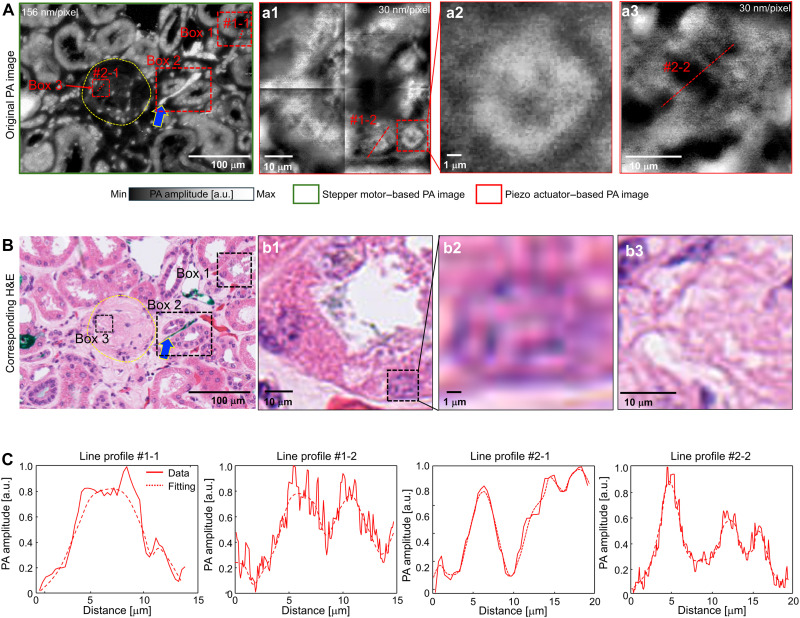
Analysis of SRUV PA images from a kidney tissue. (**A**) Original PA image of kidney tissue, with selected regions of interest (boxes 1, 2, and 3) marked for detailed analysis. (A1 to A3) Magnified views of subcellular structures from the boxed regions in (A), highlighting high-resolution nuclear features. a.u., arbitrary units. (**B**) Corresponding H&E-stained image of the same kidney tissue section, with the same regions (boxes 1, 2, and 3) marked for comparison. (B1 to B3) Enlarged views of the boxed regions in (B) illustrating nuclear and structural details observed in the histological staining. (**C**) Line profiles extracted along the dashed lines in (A) and (A1) to (A3), corresponding to subcellular structures, showing intensity variations in PA amplitude.

In human kidney tissue, histologic images acquired using SRUV-PAM (stepper motor) at the step size of 0.156 μm to reveal the cortical tissue of the kidney, showing multiple tubules in the cross section and a single glomerulus in the center (Fig. 3A). The yellow dashed circle indicates the glomerulus. A nearby section stained with H&E and imaged using a microscope shows highly similar structures (Fig. 3B). For instance, the cells lining the renal tubules highlighted in box 1 show nuclei with well-represented nuclear features and variable intensity corresponding to the DNA/hematoxylin distribution (Fig. 3, A and B). To closely examine box 1, subcellular-resolution histology-like PA images were acquired using the piezo actuator with a step size of 30 nm (Fig. 3, A1). To visualize the entire tubule, mosaic imaging was performed by acquiring images from four adjacent areas and combining them into a single image. This process introduced stitching artifacts at the boundaries between the individual tiles. To better demonstrate the resolution capability of our system, we further zoomed in on a single nucleus from the red dashed box in Fig. 3 (A1), which is shown in Fig. 3 (A2). The subcellular-resolution image in Fig. 3 (A2) reveals the structural details of the nucleus. Similarly, the corresponding H&E images in Fig. 3 (B1 and B2) allow direct comparison with the PA images. Figure 3 (B1) shows the same region as Fig. 3 (A1), where the tissue structure is visible. Likewise, Fig. 3 (B2) corresponds to the nucleus shown in Fig. 3 (A2), demonstrating a comparable structural pattern. Box 2 highlights tubules with high signal intensity associated with erythrocytes within capillaries (blue arrow) (Fig. 3, A and B). Figure 3C depicts line profiles across a single nucleus acquired with a stepper motor (line #1-1 in Fig. 3A) and a piezo actuator (line #1-2 in Fig. 3, A1), respectively. Despite representing the same nucleus, the nuclear line profile acquired with the piezo actuator distinctly shows two peaks due to the high spatial resolution, representing DNA marginations along the nuclear membrane. This enhanced visibility of nuclear margination in PAM images, compared to H&E sections, can be attributed to the fundamental differences in imaging modalities—while the H&E image represents a 5-μm-thick tissue section, PAM captures signals from a much narrower optical absorption plane, leading to a sharper delineation of nuclear structures. In addition, mesangial cells within the glomerulus show less distinct nuclear-to-cytoplasmic discrimination (box 3 in Fig. 3, A and B). In particular, we zoomed in on box 3 using the piezo actuator to examine the structure in detail (Fig. 3, A3). Line profiles #2-1 and #2-2, from the same area but acquired with the stepper motor and piezo actuator, respectively, were compared (Fig. 3D). The profile of line #2-2 reveals two clear peaks at 10 to 20 μm on the distance, distinguishing it from line #2-1. Figure 3 (B3) is the corresponding H&E image. Because the PA image section and the H&E image section do not perfectly align, there are structural differences between the two images. Notably, the H&E histology images were acquired with a 40× objective lens (NA ≈ 0.65), which is commonly used in standard histopathological evaluation. This setup provides an estimated spatial resolution of ~520 nm based on the Rayleigh criterion at a 550-nm wavelength. In contrast, our SRUV-PAM system uses a 266-nm excitation wavelength with a 0.64 NA, achieving a lateral resolution of ~240 nm. While higher-NA objectives could potentially improve the resolution of H&E imaging, our system demonstrates the capability to provide slightly enhanced image quality and subcellular detail compared to what is typically obtained in routine H&E histology.

**Fig. 4.**
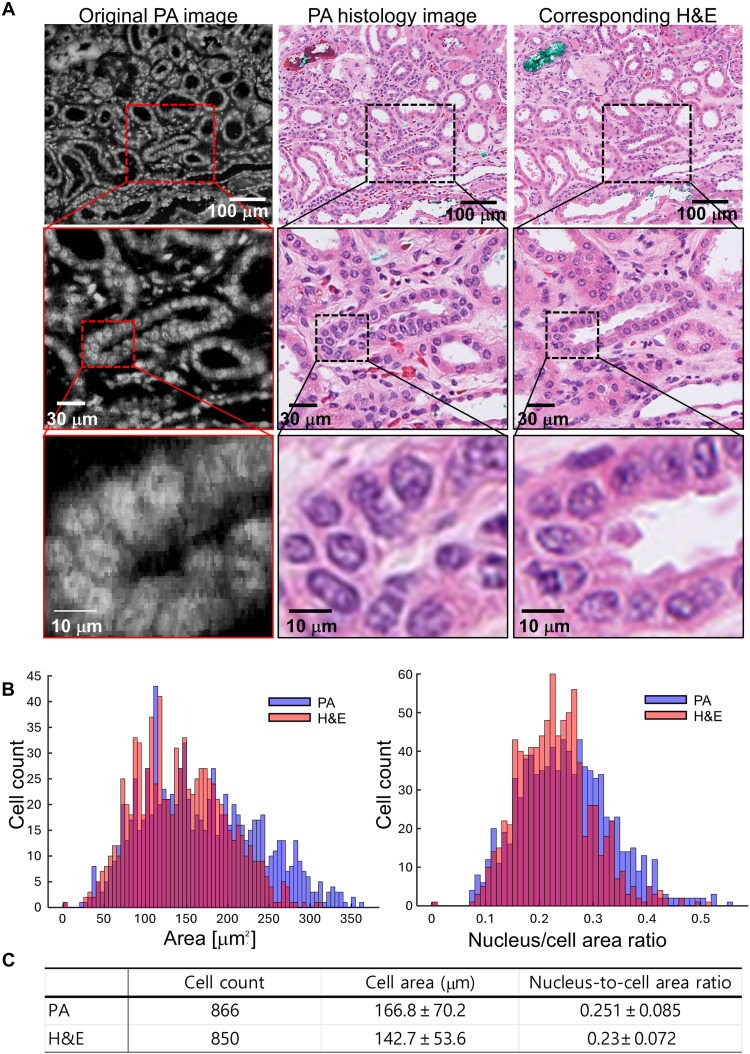
Comparative analysis of SRUV PA virtual histology images of human kidney tissues. (**A**) Original label-free PA image (left), virtually stained PA histology image (middle), and corresponding H&E-stained image (right). Enlarged insets highlight structure details in selected regions. (**B**) Histograms comparing the virtually stained PA histology image (purple) and the corresponding H&E-stained image (orange). The left histogram shows the distribution of cell areas, while the right histogram shows the distribution of nucleus-to-cell area ratios. (**C**) Quantitative comparison of cell counts, average cell areas, and nucleus-to-cell area ratios between PA and H&E images.

Figure 4 demonstrates the virtually stained PA human kidney tissue images. The original PA image captured in grayscale represents the inherent optical absorption contrast of nucleic acids in cell nuclei (the left panel in Fig. 4A). This original PA image was acquired using the stepper motor. This offers a subcellular-resolution depiction of kidney cortical tissue, highlighting the intricate arrangement of tubules and glomeruli. From the original PA image, we generated the PA virtually stained histology image using CycleGAN deep learning algorithms (center panel in Fig. 4A). This computationally enhanced image mimics traditional histological staining, providing an easily interpretable representation and differentiation of cellular and subcellular structures. Notably, the virtually stained image aligns with the structural details of the original PA image and exhibits staining that matches the corresponding H&E-stained image on the right of Fig. 4A. The H&E image, serving as the gold standard, shows cellular and extracellular components with well-established contrast, where nuclei are stained dark blue or purple due to hematoxylin, and the cytoplasm and extracellular matrix are stained in varying shades of pink, representing the eosin stain distribution. The agreement in staining patterns across these images underscores the accuracy and reliability of the virtual staining technique in replicating conventional histological results. The middle row of Fig. 4A presents enlarged images derived from the boxed regions in the original images, revealing further details. The enlarged PA image distinctly shows nuclear features with varying intensities corresponding to DNA distribution. The virtually stained PA histology image enhances the contrast of nuclear and cytoplasmic structures, closely resembling traditional histological features. The corresponding enlarged H&E-stained image provides a conventional histological perspective, confirming the structures observed in the PA images. The bottom row of Fig. 4A shows even more magnified views. In the original PA image, a single nucleus is identifiable. The virtually stained PA histology image, enhanced by pseudo-staining, provides even higher contrast, making the nucleus more distinguishable. Similarly, the corresponding H&E-stained image also depicts a single nucleus, further validating the accuracy of the virtually stained PA histology approach. The PA images, both original and virtually stained, demonstrate the capability to visualize nuclear and cytoplasmic features with high fidelity, comparable to conventional H&E staining. The quantitative analysis of cell counts, cell areas, and cell-to-nucleus area ratios is presented in Fig. 4 (B and C). The values show slight variations, which is expected because the analysis was performed on adjacent sections captured at different axial depths rather than on the exact same section. This analysis reveals a strong correlation between the virtually stained PA histology image and the corresponding H&E histology image. The achieved subcellular resolution of ~240 nm in the SRUV-PAM system enables the visualization of fine nuclear structures that are typically difficult to resolve in conventional UV-PAM systems. Notably, features such as chromatin margination, nuclear envelope irregularities, and internuclear boundaries—commonly ranging between 200 to 500 nm—are observed. This enhanced spatial detail supports high-fidelity virtual staining, as demonstrated by the morphological resemblance between virtually stained and standard H&E images. The preservation of subtle nuclear features plays a crucial role in histopathological interpretation, underscoring the importance of achieving submicron resolution in label-free imaging platforms. In addition to these morphological comparisons, we quantitatively assessed image similarity using the Fréchet Inception Distance (FID) and Kernel Inception Distance (KID). The virtually stained PA images achieved an FID of 43.64 and a KID of 0.0206 ± 0.0009, reflecting close alignment with the corresponding H&E images. These quantitative results further emphasize the reliability and high fidelity of our virtual staining method.

### SRUV-PAM for liver cancer diagnosis

**Fig. 5.**
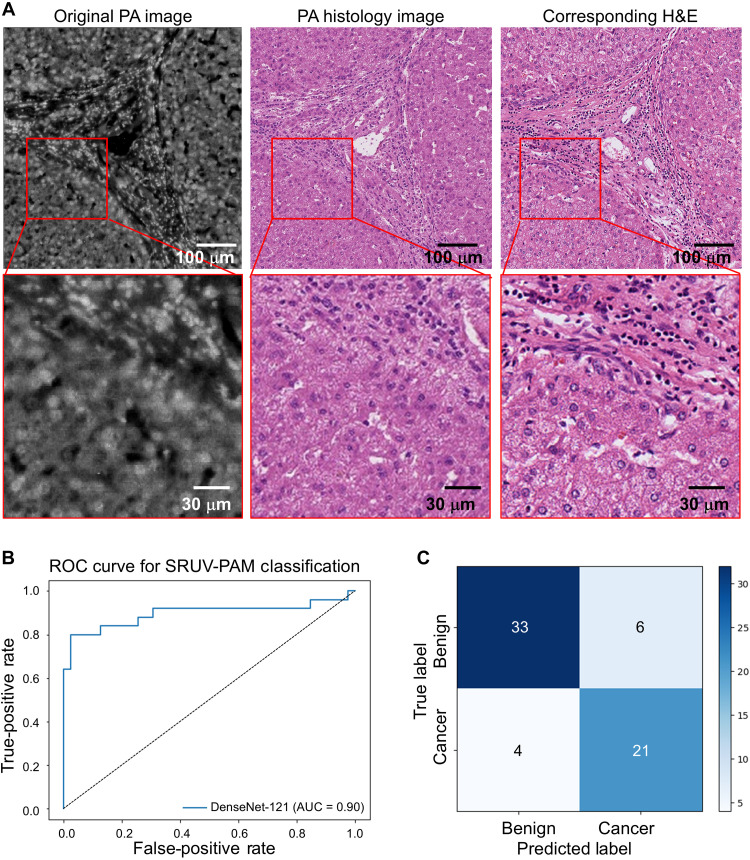
SRUV PA virtual histology images of human liver tissues and deep learning–powered diagnosis. (**A**) Original PA image (left), virtually stained PA histology image (middle), and corresponding H&E image (right). Enlarged insets highlight nodules of hepatocellular parenchyma defined by areas of expanded portal tracts with lymphoid cells. (**B** and **C**) Automated classification of liver cancer in PA images using deep learning. (B) ROC curve of the DenseNet-121 classifier for the hold-out test set, with an AUC of 0.902. (C) Confusion matrix for the hold-out test set.

Figure 5A shows the PA virtual histology images of human liver tissue. The original PA image provides a subcellular-resolution depiction of liver tissue architecture, showcasing various cellular and subcellular structures with distinct clarity. The virtually stained PA image aligns precisely with the structural details observed in the original PA image and demonstrates staining patterns that are consistent with those seen in traditional histological preparations. The virtually stained PA image effectively enhances the contrast of cellular and extracellular components, providing a clear differentiation of tissue structures. The comparison between the virtually stained PA image and the H&E-stained image demonstrates a high degree of similarity, indicating that the virtual staining process accurately replicates the conventional histological staining results. In the enlarged images at the bottom, derived from the boxed regions in the original figures, the intricate details of a hepatic liver portal tract are further revealed, differentiating vascular and bile duct structures and inflammatory cells. The enlarged original PA image (bottom left) captures nodules of hepatocellular parenchyma defined by areas of expanded portal tracts with lymphoid cells. The virtually stained PA histology image (bottom center) enhances these features, closely resembling the detailed structures seen in traditional histology (bottom right).

Using a combination of SRUV-PAM and deep learning, we performed liver cancer tissue classification by fine-tuning the DenseNet-121 model, which was pretrained on the ImageNet dataset (Fig. 5, B and C). The representative PA images of liver tissue used for model training are presented in fig. S2. The model was trained for 50 epochs using a dataset of approximately 450 SRUV-PAM image sections, each cropped to 300 μm by 300 μm. Given that SRUV-PAM images are grayscale, each section was normalized using ImageNet dataset statistics to match the distribution of the pretrained model. Hyperparameter tuning was conducted on the basis of validation performance to optimize model generalizability. The final trained model was evaluated on a hold-out test set, demonstrating a test specificity of 90% and a sensitivity of 84%, indicating a strong ability to distinguish between benign and malignant liver tissues. In addition, the model achieved an area under the receiver operating characteristic (ROC) curve of 0.902, reflecting high overall diagnostic accuracy (Fig. 5B). The confusion matrix (Fig. 5C) further illustrates classification performance: (i) Thirty-three benign tissue sections were correctly classified, while four benign sections were misclassified as cancerous. (ii) Twenty-one cancerous sections were correctly identified, with six misclassified as benign.

## DISCUSSION

In this study, we successfully demonstrated the application of an SRUV-PAM, integrated with deep learning algorithms, as a potential tool for improving cancer diagnosis. The developed SRUV-PAM system, with its 240-nm resolution, provides a notable improvement over existing UV-PAM technologies, enabling detailed subcellular imaging without the need for traditional staining methods. Deep learning–based virtual staining, implemented via CycleGAN, further enhances the interpretability of PA images, making them comparable to traditional H&E-stained slides and facilitating seamless adoption by pathologists. Using CycleGAN, we successfully generated H&E-like contrasts that highlight nuclear and tissue features, although minor artifacts were observed, likely due to the limited size of the training dataset. While emerging generative models such as diffusion-based approaches may further improve staining fidelity, our results demonstrate that even established frameworks can leverage SRUV-PAM images to support accurate diagnostic interpretation.

The diagnostic accuracy of the system was validated using a fine-tuned DenseNet-121 model, demonstrating its potential for clinical application. The model achieved a specificity of 90% and sensitivity of 84%, with an area under the ROC curve of 0.902, effectively differentiating between benign and malignant liver tissues. These results highlight the robustness of combining SRUV-PAM with deep learning–based analysis, reinforcing its viability as a noninvasive, label-free diagnostic tool.

While these findings are promising, the study primarily demonstrates the feasibility and potential of this combined imaging and deep learning approach. Further investigation and larger-scale validation studies are necessary to confirm the clinical accuracy and generalizability of the system, given the limited dataset size in this study. In addition, while we aimed to support our claim with real tissue images, we were unable to observe structures in our PA tissue images that could substantiate the system’s resolution (~240 nm). This limitation is likely due to the absence of sufficiently small structures within the imaged tissue that would allow for direct validation. We acknowledge this constraint and recognize the need for optimized imaging conditions or alternative samples in future studies. Beyond this, the system itself has technical constraints: It operates in transmission mode and relies on mechanical point-by-point scanning. These factors result in relatively long acquisition times and limit applicability to thin tissue sections. Consequently, SRUV-PAM is not yet optimized for rapid intraoperative use, real-time imaging, or thick-tissue evaluation. To address this, future extensions of the platform could incorporate flat optical components such as metalenses with extended focal depth, enabling volumetric focusing without compromising lateral resolution. We have previously demonstrated such an approach using metalenses at 532-nm excitation (*36*), and similar strategies at deep-UV wavelengths (e.g., 266 nm) could potentially broaden the applicability of SRUV-PAM to more complex tissue geometries. In our current system, acquiring a 1 mm–by–1 mm image at 0.156-μm step size and a 50-kHz repetition rate requires ~13.7 min. While this acquisition speed is not yet optimized for large-area or real-time intraoperative applications, it is well suited for proof-of-concept demonstration of subcellular-resolution imaging, particularly in use cases where high morphological detail is essential—such as digital histology or artificial intelligence–based virtual staining, as shown in Figs. 3 and 4. Because our system does not require conventional staining, the overall workflow has the potential to reduce turnaround time compared with the standard H&E protocol. This limitation can be mitigated by adopting a multiscale imaging strategy similar to standard pathology workflows. For instance, a low-magnification survey scan comparable to 4× microscopy (e.g., 10 mm by 10 mm at ~2-μm step size) would require ~25 million pixels, corresponding to only ~8 min at a 50-kHz repetition rate. An intermediate-scale scan comparable to 10× (e.g., 2 mm by 2 mm at 1-μm step size) would require ~4 million pixels, taking only ~1.3 min. A high-magnification scan comparable to 40× microscopy (e.g., 0.5 mm by 0.5 mm at 0.25-μm step size) would require ~4 million pixels, corresponding to ~1.3 min. A high-magnification scan comparable to 60× (e.g., 100 μm by 100 μm at 0.156-μm step size, as in our stepper motor mode) would require only ~0.13 min. In addition, for ultrahigh-magnification scan, the integrated piezo actuator enables acquisition of a 30 μm by 30 μm region at 30-nm positioning precision—comparable to ~100× equivalent magnification—within ~20 s (table S1). This multiscale approach keeps the total imaging time within a manageable range (on the order of 1 to 10 min, depending on the application) because high-resolution imaging is applied only to selected regions of interest. Within this framework, our PA histology system is positioned as the high-resolution modality, enabling label-free visualization of subcellular nuclear features that are critical for fine pathological assessment. To further enhance imaging throughput and expand the system’s applicability, we are actively exploring the integration of optical scanning mechanisms, such as galvanometric mirror-based beam steering, in combination with high–repetition-rate UV lasers. These upgrades, together with a multiscale imaging strategy analogous to conventional pathology practice, would allow considerable acceleration of image acquisition while preserving spatial resolution, thereby facilitating broader use in pathology-oriented workflows. Hence, our work serves as a foundation for future research that may establish this technology as a reliable tool in clinical diagnostics, particularly for liver cancer detection. In addition, a pathologist reviewed the virtually stained images and confirmed their applicability for diagnosis, further validating the potential of PA virtual histology in clinical practice.

Overall, this work represents a marked advancement in histopathology, offering a faster and more accurate alternative to traditional methods. Integrating this technology into clinical practice could revolutionize diagnostic pathology, improving patient outcomes through faster and more accurate disease detection.

## MATERIALS AND METHODS

### Label-free SRUV-PAM

The Nd:YLF Q-switched 266-nm nanosecond pulsed laser (QL266-010-O, CrystaLaser) was used as the excitation source for SRUV-PAM. A bandpass filter (FGUV5, Thorlabs) was positioned in front of the laser source to eliminate any leaked pump light. The filtered pure UV light passing through the sampler was directed to a photodetector (PDA36A, Thorlabs). This photodiode (PD)–detected light was used to compensate for the energy fluctuations in the laser. The beam passing through the sampler underwent spatial filtering through a 25-μm-diameter pinhole (PH-25, Newport). Subsequently, the beam was expanded to 15 mm using a plano-convex lens pair (LA4052-UV and LA4924-UV, Thorlabs). After expansion, the beam was precisely aligned vertically using a mirror (PF20-03-F01, Thorlabs), and an objective lens (AFL25-17-P-X-355) was used to establish the optical focus. This optical focus penetrated the 200-μm-thick sample substrate to illuminate the tissue sample. A customized highly focused UT (40-MHz center frequency) collected the PA signal generated in the tissue sample. The acquired PA signal was amplified through two preamplifiers (ZFL-500LN+, Mini-Circuit), then recorded by a data acquisition board with a sampling rate of 500 MHz (ATS9350, Alazar Technologies), and subsequently stored on a PC. To enable large-area and subcellular-resolution imaging, a 3D stepper motor scanning system (three PLS-85, Physik Instrumente) was combined with 2D piezo actuators (two Nano-OP30, Mad City Labs).

### Human tissue preparation and H&E imaging

Clinical tissue samples were obtained at the City of Hope Medical Center under an Institutional Review Board–approved protocol (IRB #21281). Tissue samples were fixed in 10% formalin, processed with standard histology techniques, embedded in paraffin blocks and sliced into 5-μm-thick sections. These sections were then mounted on either a glass or quartz slide based on the intended analysis. The sections on glass slides were subjected to standard H&E staining before being cover slipped. H&E histology slides were imaged using a standard optical microscope or a digital whole-slide scanning microscope, specifically the Leica Aperio AT2, with a ×40 objective. In contrast, the sections mounted on quartz, a UV-transparent material, were not stained and scanned directly using SRUV-PAM.

### Pseudo-staining via neural networks

The generators in the network are designed as residual networks, starting with an input convolutional layer, followed by two convolutional layers paired with downsampling blocks. This is succeeded by nine residual network blocks, then two convolutional layers paired with upsampling blocks, and concluding with an output convolutional layer. Instance normalization and rectified linear unit (ReLU) layers are used after each convolutional layer to maintain stability and improve performance.

For the discriminator, a PatchGAN architecture is used, which operates by classifying image patches of 70 by 70 pixels to determine whether they are real or generated. This specific patch size is chosen to balance, maintaining high spatial frequency fidelity and preventing tiling artifacts. Within the discriminator, each convolutional layer is followed by instance normalization and leaky ReLU (lReLU) layers, where lReLU(*x*) is defined as max(0.2*x*, *x*), adding nonlinearity and improving the model’s ability to learn complex features.

In addition, to enhance shift invariance, both the generators and discriminators incorporate antialias downsampling and upsampling layers. This approach helps in preserving the integrity of the spatial details across different scales within the network, leading to more consistent and accurate image generation and classification.

### Tumor diagnosis via deep learning

Whole-slide SRUV-PAM images from 12 patients were cropped into a regular grid of 587 nonoverlapping sections, each measuring 1024 by 1024 pixels (corresponding to 300 μm by 300 μm). This section size was chosen to balance information content and dataset diversity: Smaller sections lacked sufficient structural features for reliable classification, whereas larger sections substantially reduced the number of available training samples. To ensure data quality, sections that were out of focus were excluded, and bright spot removal was performed as described in Materials and Methods. On the basis of the pathologist’s annotations, tumor and benign regions were separately processed to construct distinct datasets (fig. S3). Each section used contains only benign or only tumor tissue. The dataset was split by slide to contain ~80/10/10% of sections for training, validation, and testing. A detailed breakdown of the training, validation, and testing set sizes is included in table S2. To remove bright spot outliers, sections were normalized on the basis of the minimum and maximum pixel values calculated after median filtering with a 20 × 20 filter. One-channel grayscale sections were converted into three-channel RGB by setting each channel to the grayscale values, before being resized into 3 by 224 by 224 images. Dataset augmentation was performed on the training set using random vertical and horizontal flips, rotations, and translations. All input sections were normalized using ImageNet statistics of mean (0.485, 0.456, and 0.406) and SD (0.229, 0.224, and 0.225) for the RGB channels, respectively.

The DenseNet-121 model consists of four dense blocks with transition layers and feature map concatenation in between blocks, with final output layers modified for binary classification. The model was initialized with ImageNet pretrained weights and further trained on the SRUV-PAM training dataset of 453 cropped sections, with all weights updated. Training was performed for up to 50 epochs using a cross-entropy loss function and an AdamW optimizer with batch size of 8, base learning rate of 1 × 10^–6^, betas (0.9 and 0.999), and weight decay of 0.01. The learning rate was reduced using a cosine annealing schedule with three linear warm-up epochs, *T*_max = 50, and no floor, with an ablation study shown in table S3. Early stopping was used on the basis of validation set performance to avoid overfitting. Model hyperparameters such as weight decay, initial decay rate, and dropout rate were tuned. Other model architectures such as VGG-16, ResNet-18, ResNet-50, and DenseNet-161 were also trained and evaluated (fig. S4 and table S4).

Model training was performed using Python 3.7.16 and PyTorch 1.7.1 (CUDA 12.8) on an NVIDIA A100 graphics processing unit (GPU). Training for 50 epochs took ~30 min on a single GPU. The best model was selected on the basis of model performance on the validation set of 70 SRUV-PAM sections. Ground truth labels for the SRUV-PAM sections were acquired from trained pathologists. The sensitivity and specificity of the trained classifier were evaluated on the hold-out test set of 64 sections, and the ROC curve was constructed using the hold-out test set with different classification thresholds.
