# Supplementary material for: Rapid cancer diagnosis using deep learning–powered label-free subcellular-resolution photoacoustic histology
Source: Sci Adv. 2025 Nov 21;11(47):eadz1820. doi: 10.1126/sciadv.adz1820 (PMC12637292; doi:10.1126/sciadv.adz1820)
Supplement: Supplementary file 1 — Figs. S1 to S4 Tables S1 to S4 [file sciadv.adz1820_sm.pdf]

Science Advances
AAAS

Supplementary Materials for

Rapid cancer diagnosis using deep learning–powered label-free subcellular-resolution photoacoustic histology

Byullee Park *et al.*

Corresponding author: Massimo D'Apuzzo, MDapuzzo@coh.org; Lihong V. Wang, LVW@caltech.edu

Sci. Adv. **11**, eadz1820 (2025)
DOI: 10.1126/sciadv.adz1820

This PDF file includes:

Figs. S1 to S4
Tables S1 to S4

Figure S1. Correction of piezo motor-induced scanning distortion. (A) Photoacoustic images of a carbon fiber sample acquired using a piezo motor scanner. The left three panels show raw images from different scanning positions. Due to the non-uniform velocity of the piezo motor—particularly the acceleration and deceleration phases—distorted signals are observed, especially in the central regions where the scanning speed is higher, resulting in narrow or faint signals. The right panel displays the image after applying software-based correction to compensate for these velocity-dependent artifacts. **(B)** Corresponding intensity profile plots before and after correction. PA, photoacoustic.

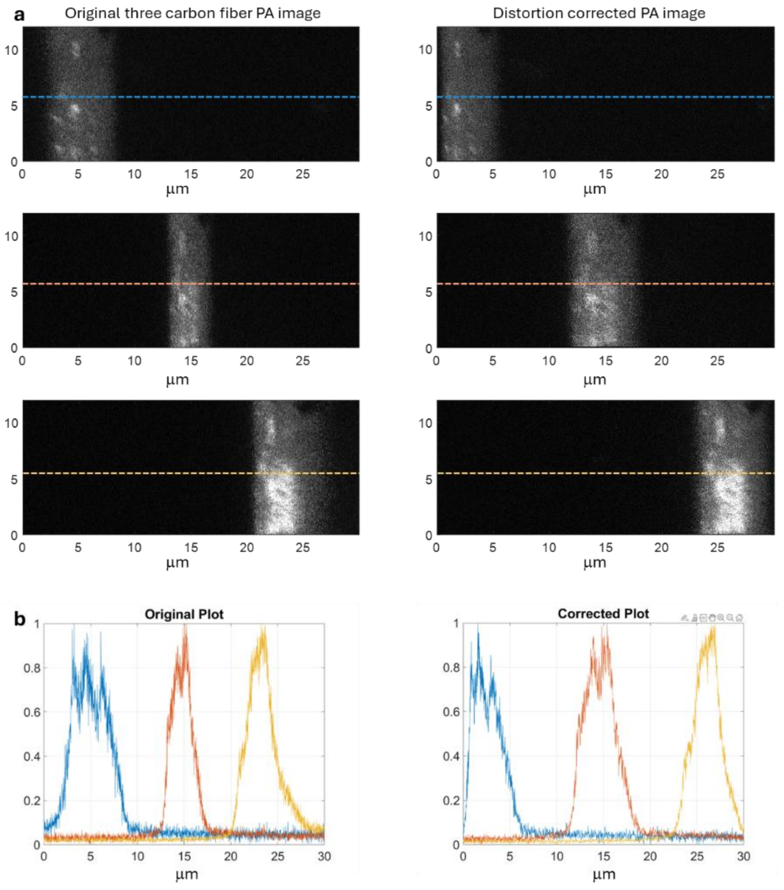

Figure S2. **Representative photoacoustic images of liver tissue for tumor diagnosis using deep learning**. Two benign cases (left column) and two malignant cases (right column).

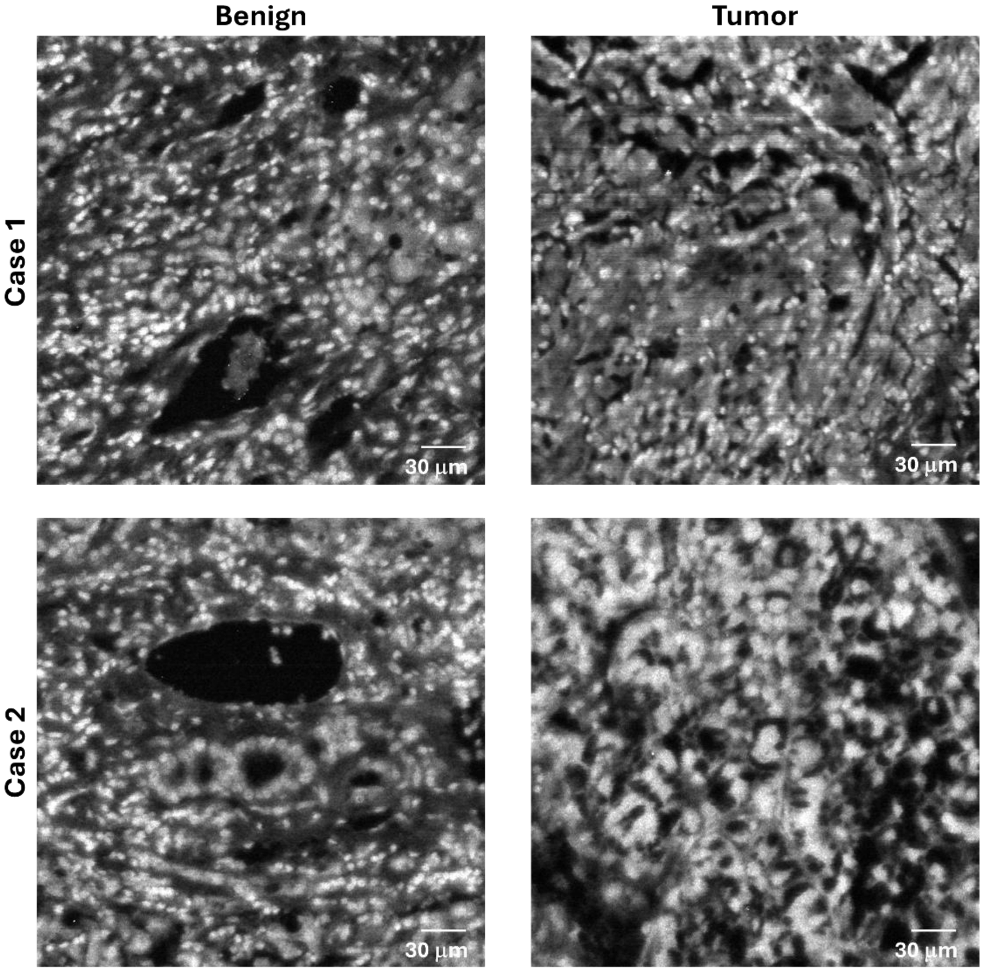

Figure S3. **Example of pathologist annotations on whole-slide H&E image, delineating tumor regions (red) and benign regions (yellow).** H&E, hematoxylin and eosin.

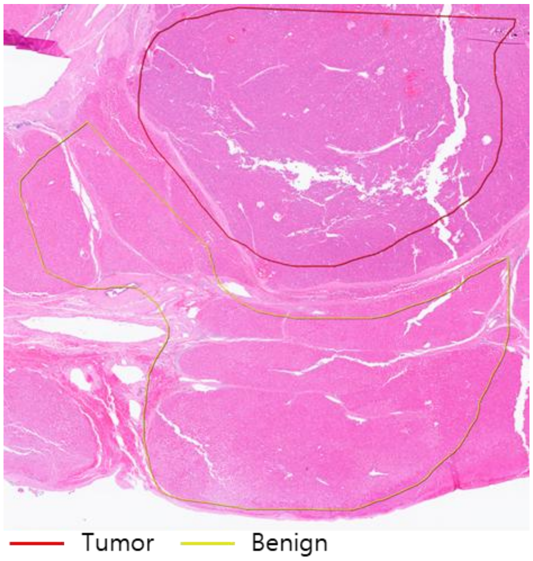

Figure S4. Additional classification model ROC curves and confusion matrices. ROC, receiver operating characteristic; AUC, area under the curve.

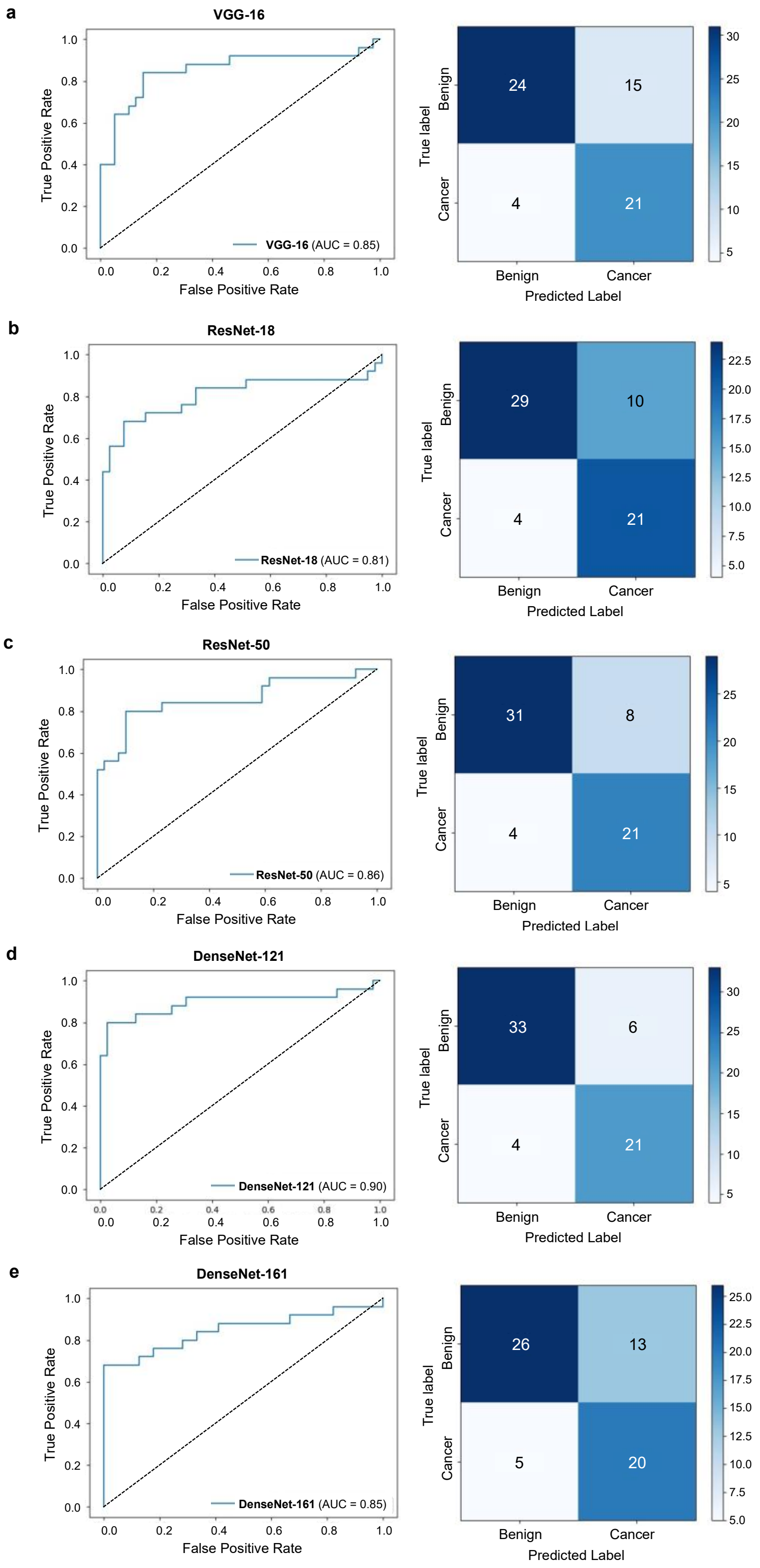

Table S1. Estimated acquisition times of the proposed SRUV-PAM system at 50 kHz under different field-of-view and resolution settings, modeled after standard pathology magnifications. SRUV-PAM, subcellular-resolution ultraviolet photoacoustic microscopy.

Imaging Mode (Analogous to Pathology)	Field of View	Pixel Resolution (step size)	Total Pixels (million)	Acquisition Time @ 50 kHz
Low-magnification (～4×)	$10 \times 10\ mm^2$	$2.0\ \mu m$	25	~8.3 min
Intermediate magnification (～10×)	$2 \times 2\ mm^2$	$1.0\ \mu m$	4	~1.3 min
High magnification (～40×)	$0.5 \times 0.5\ mm^2$	$0.25\ \mu m$	4	~1.3 min
High magnification (～60×, stepper motor)	$100 \times 100\ \mu m^2$	$0.156\ \mu m$	0.41	~0.17 min
Ultra-high magnification (～100× equivalent, piezo actuator)	$30 \times 30\ \mu m^2$	30 nm	1.0	~20 sec

Table S2. Training, validation, and testing set sizes.

**Set**	**Benign Sections**	**Tumor Sections**	**Total Sections**
Training	260	193	453
Validation	36	34	70
Testing	39	25	64
Total	335	252	587

Table S3. Learning rate tuning in ablation study for the DenseNet-121 model. The AdamW optimizer with a smaller base learning rate performs better at reducing overfitting. A cosine annealing schedule with 3 linear warm-up epochs and no floor is used during model training, and the loss function is a cross-entropy loss. The Dense-Net 121 model is pre-trained on the ImageNet dataset and fine-tuned on SRUV-PAM image sections, with all model weights updated during training.

Optimizer	Base Learning Rate	Training Loss	Training AUC	Validation Loss	Validation AUC	Testing AUC
**AdamW**	1e-4	0.09	0.99	3.31	0.79	0.70
(betas = (0.9, 0.999),	1e-5	0.21	0.97	0.75	0.77	0.80
weight_decay = 1e-2)	1e-6	0.49	0.91	0.58	0.79	0.90

Table S4. Additional classification model metrics. All models are pre-trained on the ImageNet dataset before fine-tuning on SRUV-PAM image sections. SRUV-PAM, subcellular-resolution ultraviolet photoacoustic microscopy; AUC, area under the curve.

Model	Training AUC	Validation AUC	Testing AUC	Testing Accuracy	Testing Sensitivity	Testing Specificity
**VGG-16**	0.81	0.73	0.85	0.81	0.84	0.79
**ResNet-18**	0.98	0.78	0.81	0.70	0.80	0.64
**ResNet-50**	0.99	0.79	0.86	0.78	0.84	0.74
**DenseNet-121**	0.91	0.79	0.90	0.88	0.84	0.90
**DenseNet-161**	0.97	0.78	0.85	0.72	0.80	0.67
